# The relationship between resident burnout and safety-related and acceptability-related quality of healthcare: a systematic literature review

**DOI:** 10.1186/s12909-017-1040-y

**Published:** 2017-11-09

**Authors:** Carolyn S. Dewa, Desmond Loong, Sarah Bonato, Lucy Trojanowski, Margaret Rea

**Affiliations:** 1 Department of Psychiatry and Behavioral Sciences, University of California, Davis, 2230 Stockton Boulevard, Sacramento, CA 95817 USA; 2 Centre for Research on Employment and Workplace Health, Centre for Addiction and Mental Health, 33 Russell Street, Toronto, M5S 2S1 Canada; 3 Library Services, Centre for Addiction and Mental Health, 33 Russell Street, Toronto, M5S 2S1 Canada; 4 School of Medicine and Betty Irene Moore School of Nursing, UC Davis Health System, 4610 X Street, Education Building, 4th floor, Room 4101B, Sacramento, CA 95817 USA

**Keywords:** Burnout, Residents, Quality of care

## Abstract

**Background:**

There has been increasing interest in examining the relationship between physician wellbeing and quality of patient care. However, few reviews have specifically focused on resident burnout and quality of patient care. The purpose of this systematic literature review of the current scientific literature is to address the question, “How does resident burnout affect the quality of healthcare related to the dimensions of acceptability and safety?”

**Methods:**

This systematic literature review uses a multi-step screening process of publicly available peer-reviewed studies from five electronic databases: (1) *Medline Current*, (2) *Medline In-process*, (3) *PsycINFO*, (4) *Embase,* and (5) *Web of Science*.

**Results:**

The electronic literature search resulted in the identification of 4638 unique citations. Of these, 10 articles were included in the review. Studies were assessed for risk of bias. Of the 10 studies that met the inclusion criteria, eight were conducted in the US, one in The Netherlands, and one in Mexico. Eight of the 10 studies focused on patient safety. The results of these included studies suggest there is moderate evidence that burnout is associated with patient safety (i.e., resident self-perceived medical errors and sub-optimal care). There is less evidence that specific dimensions of burnout are related to acceptability (i.e., quality of care, communication with patients).

**Conclusions:**

The results of this systematic literature review suggest a relationship between patient safety and burnout. These results potentially have important implications for the medical training milieu because residents are still in training and at the same time are asked to teach students. The results also indicate a need for more evidence-based interventions that support continued research examining quality of care measures, especially as they relate to acceptability.

**Electronic supplementary material:**

The online version of this article (10.1186/s12909-017-1040-y) contains supplementary material, which is available to authorized users.

## Background

Reports from around the world indicate that 27% to 75% of residents regardless of specialty experience burnout [1]. Burnout has been conceptualized as a syndrome consisting of three dimensions: Emotional Exhaustion (EE), Depersonalization (DP) and low Personal Accomplishment (PA) [2]. There is evidence that practicing physicians who experience burnout are also affected by lower personal well-being including low job satisfaction [3–5] and decreased mental health [6].

There is concern that resident training may contribute to burnout. For example, a national survey of US medical students and residents found that residents were significantly more likely to experience burnout with 44% of resident respondents reporting high levels of EE and 51% experiencing high levels of DP [7]. In their study, Ripp et al. [8] found that at the beginning of their first post-graduate year, 14% of study participants experienced burnout. By the end of that year, the proportion experiencing burnout increased to 50%. These results suggest that training contributes to burnout in residents. At the same time, burnout has also been linked to decreased cognitive functioning [9]. It is also during this period that people are being trained to practice independently. Thus, residents are potentially in situations in which they seek to learn new skills while being exposed to the risk of burnout and decreased cognitive functioning. This raises the question of how burnout can affect performance of those skills. If burnout impedes acquiring new skills, it can be counterproductive to resident training. One step toward understanding the relationship in residents is to examine the association between resident burnout and quality of care.

The purpose of this systematic literature review is to address the question, “How does resident burnout affect the quality of healthcare related to the dimensions of acceptability and safety?” There are six quality of care dimensions: effectiveness, efficiency, accessibility, equitability, acceptability, and safety [10, 11]. In this review, we focus on the two dimensions of quality – acceptability (i.e., patient satisfaction, perceived quality of care, and communication) and safety (i.e., minimizing risks or harm to patients). These two dimensions were chosen because they reflect the quality of physician-patient interactions [12]. That is, if a clinician’s wellbeing is compromised, their patient interactions may also be negatively affected [13]. In contrast, effectiveness, efficiency, accessibility, and equitability reflect the systems (i.e., infrastructure, information technology, payment policies) in which practice is conducted [10]. The focus on the quality of care dimensions related to the physician-patient relationship can provide important additional information about how the residency experience affects patients who are treated by residents.

There has been growing interest in examining the relationship between physician wellbeing and quality of patient care. Although the World Health Organization [11] and the US Institute of Medicine (IOM) [10] identify six dimensions of quality of healthcare, attention has focused on the dimension of patient safety. Recently, there have been three published reviews examining the relationship between clinician and physician wellbeing and patient safety [14–16]. There has been one systematic review on physician wellbeing and quality of patient care [17]. However, each of these published reviews answered different questions from the one we address in our review. For example, none focused specifically on residents. Although residents are part of the group providing clinical care, because they are still in training, their role is different from physicians who have completed training. Thus, by combining the groups, there is the potential to overlook experiences that may be unique to residents.

As a result of the differences in foci, the already published reviews employed different search strategies and inclusion/exclusion criteria. Consequently, they included different articles from ours. For example, Hall et al.’s [15] review does not include five articles that are included in our systematic review. We included three articles related to acceptability and two articles related to patient safety that were not included in Hall et al.’s [15] review. Furthermore, between our two reviews, there are only seven papers that overlap; one is on acceptability and six on patient safety. In comparison to de Jong et al. [14], our review has four articles that are unique to our systematic review; three are related to acceptability and one to patient safety. Scheepers et al. [17] includes one of the papers in our review. None of the articles included in our review were included in Williams and Skinner’s [16]. Thus, our review includes papers that have not been considered together to look at the impact of resident burnout on quality of care.

Furthermore, only one [17] of the published reviews include the acceptability dimension of quality of care. Yet, in addition to patient safety, this dimension is associated with the quality of interactions between providers and patients. Acceptability is also encompassed under the IOM’s [10] quality dimension of patient-centered care which is distinguished by care that is respectful, responsive to patient preferences, needs, and values [10]. In the healthcare setting, the physician-patient interaction is a fundamental interaction [12, 16]. The quality of these interactions is reflected in the communication quality and physician empathy which in turn contribute to perceived quality of care and patient satisfaction [10, 12] and ultimately, to the acceptability of care. The quality of the physician-patient interaction also affects the partnership between the physician and patient. The strength of this collaboration supports better patient outcomes [12]. In their role as care providers, residents also impact quality of patient care through the quality of their interactions with patients.

## Methods

The *Preferred Reporting Items for Systematic Reviews and Meta-Analyses* (PRISMA) guidelines [18] were followed to conduct the systematic review of the literature (see Additional file 1 for PRISMA checklist). Ethics board review was not sought because this review used only publically available information.

### Information sources

There were five databases searched: (1) *Medline Current* (an index of biomedical research and clinical sciences journal articles); (2) *Medline In-Process* (an index of biomedical research and clinical sciences journal articles awaiting to be indexed into *Medline Current*); (3) *PsycINFO* (an index of journal articles, books, chapters, and dissertations in psychology, social sciences, behavioral sciences, and health sciences); (4) *Embase* (an index of biomedical research, biomedical abstracts, drug and medical device conferences); and (5) *Web of Science* (an index of journal articles, editorially selected books and conference proceedings in life sciences and biomedical research).

### Search strategy

With the team’s professional health science librarian (SB), search strategies were developed and tailored for each database (see Additional file 2 for search strategy and keywords). Our search strategy reflected the Peer Review of Electronic Search Strategies (PRESS) 2015 Guidelines [19]. The searches were conducted between August 2015 and October 2015. The OVID platform was used to search *Medline Current*, *Medline In-Process*, *PsycINFO*, and *Embase*. *Web of Science* was searched using the Thomson Reuters search interface. The search period covered January 2002 to September 2015 and all searches were limited to English language journals. The time frame was chosen to represent the healthcare environments in which residents are currently being trained and practicing. The searches sought to identify articles about medical residents working in civilian settings regardless of specialty. A broad search strategy was employed to increase the likelihood that all studies on resident burnout would be found. That is, the search strategy did not seek to exclude physicians who were not residents. The reference lists of all accepted full-text articles were hand searched.

### Screening process

Relevant articles were identified using a multi-step screening process that involved two independent reviewers (CSD and LT) at each step. In Step One, titles were screened for relevance. In Step Two, the abstracts of the remaining articles were screened. The final step of the screening process involved screening the full text of all articles that passed Steps One and Two. Papers for which there was insufficient information in the title and abstract to determine relevancy were screened during the full-text screening stage. The inter-rater reliability corrected for chance [20] between CSD and LT was κ = 0.96. Before moving onto each stage, disagreements were discussed until consensus was reached.

For this review, burnout was defined as a syndrome of emotional exhaustion, cynicism (depersonalization) and reduced feelings of personal accomplishment related to work [2]. Quality of care related to acceptability was defined by measures of patient satisfaction, perceived quality of care, resident communication with patients, and resident attitudes towards patients. In addition, safety was defined by measures of medical errors.

Study inclusion criteria were:

1. Studies reported quality of care outcomes related to acceptability (i.e., satisfaction, patient preferences, and collaborative decision making) or safety (i.e., minimizing risks or harm to patients),
2. The sample population was comprised of residents working in civilian settings regardless of specialty,
3. Burnout was assessed based on a validated measure. For our review, a validated measure was defined as a measure for which there was evidence of its validity and reliability. The psychometric properties could either be provided in the text of the paper or with a reference to another paper, and
4. Paper reports original research.

Exclusion criteria were:

1. The study sample was comprised only of non-residents,
2. The study did not examine the relationship between burnout and one of the two quality of care dimensions,
3. A validated measure of burnout was not used (i.e., there was no evidence that the psychometric properties of the measure had been evaluated), and
4. The paper was a review article or commentary.

Articles for which there was disagreement regarding inclusion were discussed after each phase until consensus was reached.

### Risk of bias assessment

In this review, we used the Cochrane Handbook’s [21] definition of bias. It is defined as, “a systematic error, or deviation from the truth, in results or inferences.” The Cochrane Handbook [21] distinguishes quality from bias in that a study may have been “performed to the highest standards possible yet still have an important risk of bias.” Thus, rather than quality, our assessment focuses on risk of bias. To assess the risk of bias in observational studies (such as those that were included in this review), Sanderson et al. [22] recommend the use of a transparent checklist that concentrates on the “few, principal, and potential sources of bias in a study’s findings”. They assert checklists should include items that account for: (1) the appropriate selection of participants, (2) appropriate measurement of variables, and (3) appropriate control of confounding. In accordance with their recommendations and the Strengthening of Observational Studies in Epidemiology (STROBE) [23] criteria, we used a 9-item checklist based on Lagerveld et al. [24] that included the following criteria:

1. Study population is well described to facilitate understanding about the generalizability of the results based on the study sample (e.g., age, sex, location of the study, physician specialty, practice location),
2. Data collection methods that address the risk of bias are described,
3. Participation/response rate was at least 50% on average,
4. Used a validated outcome measure/process,
5. Statistical method was appropriate for the question being answered,
6. Statistical significance of associations were tested and reported,
7. Study controlled for relevant confounding factors - at least one confounder such as sex or age was considered in the analyses,
8. Resident matched with patient rather than matching the data from the Unit in which the resident was practicing and patients that were treated by the Unit, and
9. Longitudinal data were used.

Each item was scored “1” if the criterion had been met and “0” otherwise. Each article could achieve a maximum score of 9. Based on their total score, articles were categorized either as low risk of bias (9–8 points), moderate risk of bias (7–5 points), or high risk of bias (1–4 points). The cut-offs were based on the US academic grading system such that missing 10%–20% of points was equivalent to excellence/good (i.e., low risk of bias), missing 30%–50% is equivalent to average/fair (i.e., moderate risk of bias), and missing more than 50% is equivalent to poor (i.e., high risk of bias).

## Results

### Article inclusion and exclusion results

**Fig. 1:**
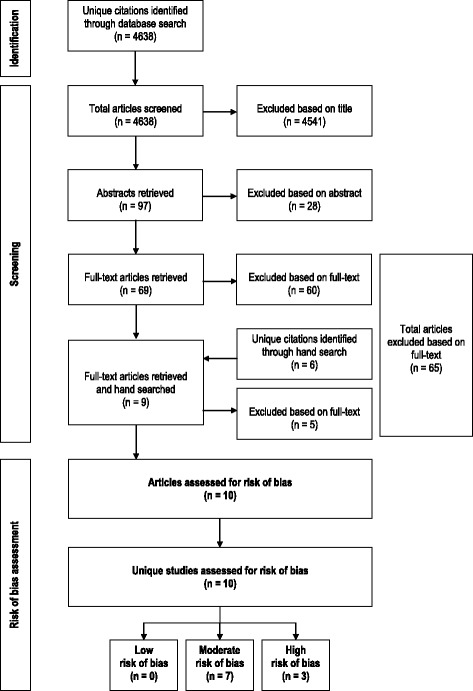
Flowchart of literature search results and the selection process of accepted/rejected articles

The electronic literature search resulted in the identification of 4638 unique citations (Fig. 1). Based on the title review, 4541 citations were excluded; 97 articles were screened in Step Two during the abstract review stage. During the abstract review, another 28 citations were excluded; this left 69 articles that were reviewed during the full-text review stage. Reasons for article exclusion at full-text review were: (1) not a relevant outcome (*n* = 11), (2) sample not comprised of residents/cannot distinguish residents as a group separate from other groups (*n* = 16), (3) it was not original research (*n* = 21), (4) burnout not measured with a validated instrument (n = 1), and (5) not published in a peer-reviewed journal (*n* = 9). After the full-text review, nine articles remained and their reference lists were hand searched for relevant studies. The hand search identified six additional citations; five were excluded (the reasons for exclusion are included in the counts above) and one was accepted at full-text review.

### Risk of bias assessment results

**Fig. 2:**
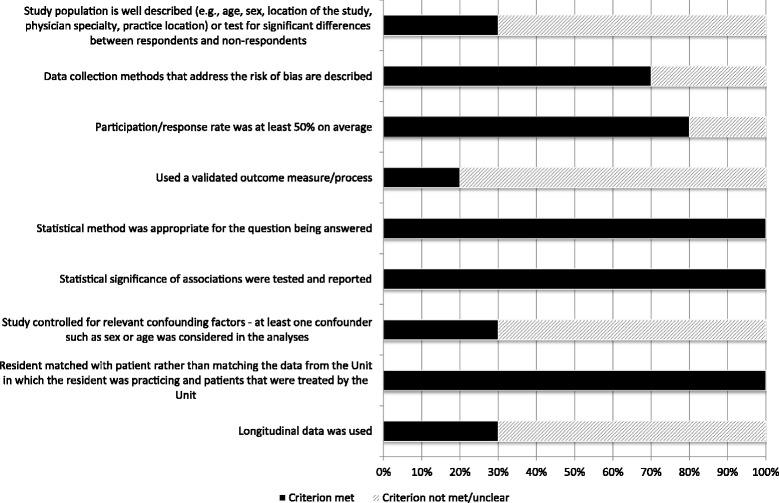
Summary of risk of bias assessment results across accepted studies

Our assessment indicated seven studies were of moderate risk of bias, and three were of high risk of bias. Figure 2 illustrates the strengths and limitations of each study. In terms of factors that reduce risk of bias, all included studies employed appropriate statistical tests, reported the results of the statistical testing, and matched residents with patients. Three studies either described the population from which the study sample was drawn [25] or tested for significant differences between study respondent and non-respondent groups [26, 27]. Three studies used longitudinal data [27–29]. A major limitation of the included studies was not controlling for possible confounding factors in the statistical analyses [25, 27–32] (see Additional file 3 for the complete Risk of Bias Assessment Checklist).

### Overview of the studies

Of the 10 studies that met the inclusion criteria, eight were conducted in the US, one in The Netherlands, and one in Mexico (Table 1).

**Table 1:** Description of the Studies

Author(s)	Study Population	Sample Description	Burnout Measure	Quality of Care Outcome Measure
Beckman et al. (2012) [26] USA	Internal Medicine residents at one institution Jun 2009 – Aug 2010 Response rate: 64.7% No significant difference between responders versus non-responders with respect to year of training, age, sex, program type, or US Medical Licensing Examination Step 1 or 2 Clinical Knowledge scores	*n* = 202 Internal Medicine residents Male = 57.4% Female = 42.6% Age: 24–30 yrs. = 85.5% ≥ 31 yrs. = 14.5% Year of training: PGY 1 = 72.8% PGY 2 = 13.9% PGY 3 = 13.4%	22-item Maslach Burnout Inventory Burnout defined as high scores on either DP or EE	6-items from the Mini-Clinical Evaluation Exercise (CEX) completed by peers, senior residents and non-physician professionals for each resident: • Desirability as a physician for one of your family • Desirability as a future co-worker or team member • Effectiveness and completeness of sign-outs • Coverage of cross-over issues and completeness of tasks while on call • Demonstrates empathy and compassion for patients • Communication skills with patients, family, allied health, and other providers
Block et al. (2013) [30] USA	1st year Internal Medicine residents from three Internal Medicine residency programs in Baltimore May – Jun 2011 Response rate: 72%	*n* = 55 Internal Medicine residents Male = 53% Female = 47% Mean age: 29 yrs. ± 3	6 items from the Maslach Burnout Inventory Burnout was scored into 3 categories based on tertiles of the sample scale scores: low, medium, high	Details about the items from the questionnaires used were not given. From the paper, error items included: • Make errors due to fatigue • Make errors due to workload • Forget to order med • Forget to convey info
de Oliveira et al. (2013) [31] USA	Residents in Anesthesiology departments in the American Society of Anesthesiologists directory Response rate: 54%	*n* = 1430 Anesthesiology residents Male: 57% Female: 43% Age: ≤ 30 yrs. = 54% > 30 yrs. = 46% Year of training: 1st-2nd yr. = 51% 3rd – 4th yr. = 49%	12 items from the Maslach Burnout Inventory: 3 from DP domain, 5 from EE domain, and 4 from PA domain High risk of burnout defined as having moderate high or high burnout sub-scale scores in 2 or more sub-scales	7-items adapted three studies (Prins et al. [32]; West et al. [27]; Shanafelt et al. [33]): • I make mistakes without negative consequences to my patients • I perform procedures without appropriate training • I make mistakes with negative consequences to my patients • I fall short in the quality of care I provide to my patients • I do not have enough time or attention for my patients • I have made medication errors involving the wrong drug or dose in the last year • I do not monitor the patient in the operating room as closely as I should
Fahrenkopf et al. (2008) [25] USA	Residents in Pediatrics and medicine-pediatrics at three institutions Response rate: 50% No significant difference between responders versus non-responders with respect to year of training, age, and sex	*n* = 123 Pediatric residents Male = 30% Female = 70% Age: < 30 yrs. = 62% ≥ 30 yrs. = 38% Year of training: PGY 1 = 33% PGY 2 = 34% PGY 3 = 33%	22-item Maslach Burnout Inventory medical staff version High burnout if combined score for EE (≥ 27) and DP (≥ 10)	Trained reviewers conduct daily review of charts and medication orders for all patients on the survey wards and a review of solicited and voluntary error reports by staff. Intensive care units and ambulatory settings excluded. Abstracted information categorized by trained reviewers as: (1) preventable adverse event, (2) non-preventable adverse event, (3) potential adverse event, (4) error with little potential for harm
Passalacqua and Segrin (2012) [28] USA	Internal medicine residents in one internal medicine program in the Southwest Response rate: not reported Measures taken pre and post long-call shift	*n* = 93 internal medicine residents Male: 62 Female: 30 Mean age: 29.6 ± 3.2 yrs	22-item Maslach Burnout Inventory Scored using a sum of all items	Adaptation of a 13-item patient-centered communication scale (Wanzer et al. [38]). Original scale designed for nurses and patients to evaluate physician-centered communication. Adaptation asked physicians to think of their shift from midnight until end of shift and report on selected behavior using a 5-point scale from 1 (never) to 5 (very often). State empathy assessed using an adaptation of Tsang and Stanford’s [40] 8-item state empathy scale. Adaptation asked statements regarding emotions toward a particular person using a 7-point scale from 1 (strongly disagree) to 7 (strongly agree)
Prins et al. (2009) [32] The Netherlands	All residents in training on October 1, 2005 Response rate: 41.1%	*n* = 2115 residents Specialties: General surgery: 8.0% Surgical specialties: 12.8% Internal medicine: 13.8% Medical specialties: 23.5% Obstetrics/gynecology: 5.9% Pediatrics: 7.7% Psychiatry: 11.5% Supportive specialties: 16.8% Male: 39% Female: 61% Mean age: 31.5 ± 3.5 yrs. Mean years in training: 3.1 ± 1.5 yrs.	20-item Utrecht Burn-Out Scale (UBOS)/Maslach Burnout Inventory for Health and Social Services Scored using UBOS manual cut-offs to diagnose moderate and severe burnout	Based on six-items from a previous study (Shanafelt et al. [33]) with a two factor solution: Action/experience error: • I make mistakes without negative consequences for the patient • I perform procedures for which I am not properly trained • I make mistakes that have negative consequences for the patient Errors due to lack of time: • I discharge patients later because my workload is too heavy • I fall short in the quality of care I provide • I do not have enough time for my patients
Shanafelt et al. (2002) [33] USA	All internal medicine residents in hospitals affiliated with one University Feb 2001 Response rate: 76% There was a decreasing response rate by residency year	*n* = 115 internal medicine residents Male: 47% Female: 53% Year of training: PGY 1 = 48% PGY 2 = 30% PGY 3 = 23%	22-item Maslach Burnout Inventory Scored each of the three domains based on terciles of a published study of 1104 medical professionals: low, medium, high Burnout defined as high score on DP or EE	Self-reported frequency of suboptimal patient care practices: • I found myself discharging patients to make the service ‘manageable’ because the team was so busy • I did not fully discuss treatment options or answer a patient’s questions • I made treatment or medication errors that were not due to a lack of knowledge or inexperience • I ordered restraints or medication for an agitated patient without evaluating him or her • I did not perform a diagnostic test because of desire to discharge a patient Self-reported frequency of suboptimal patient care attitudes: • I paid little attention to the social or personal impact of an illness on a patient • I had little emotional reaction to the death of one of my patients • I felt guilty about how I treated one of my patients from a humanitarian standpoint Frequency: never, once, several times per year, monthly, weekly Summary measures: • Suboptimal patient care practices at least monthly • Suboptimal patient care practices at least weekly
Toral-Villanueva et al. (2009) [34] Mexico	Junior doctors at three hospitals in the Mexican Health System Sep 2003 – Jan 2004 Response rate: 65%	*n* = 312 junior doctors On-the-job seniority: 28 ± 17.6 mths Male: 57% Female: 42% Mean age: 28 ± 2.5 yrs	22-item Maslach Burnout Inventory validated in Spanish. Scored using low, moderate, high cutoffs Burnout defined as high scores for either DP(≥ 10) or EE (≥ 27)	Used Shanafelt et al. [33] items and scoring methods (see above)
West et al. (2006) [27] USA	Internal medicine residents at one institution in academic years 2003–2004, 2004–2005, and 2005–2006 Data collected up to May 2006 Residents surveyed every 3-months throughout training Response rate: 84% No significant difference between responders versus non-responders with respect to age, sex and program type	*n* = 184 internal medicine residents Male: 51.1% Female: 35.9% Age: ≤ 30 yrs. = 70.1% > 30 yrs. = 16.3%	22-item Maslach Burnout Inventory Burnout defined as high DP, high EE, and low PA scores	Single question asked every 3 months, “Are you concerned that you have made any major medical errors in the last 3 months?”
West et al. (2009) [29] USA	Internal medicine residents at one institution in academic years in the residency program from July 2003 – Feb 2009 Residents surveyed every 3 months throughout their training beginning in 2003 Response rate: 88.3% No significant difference between responders versus non-responders with respect to age, sex and program type	*n* = 380 internal medicine residents Male: 62.1% Female: 37.9% Age: ≤ 30 yrs. = 63.2% > 30 yrs. = 14.7%	Maslach Burnout Inventory Burnout defined as high DP, high EE, and low PA scores	Single question asked every 3 months, “Are you concerned that you have made any major medical errors in the last 3 months?”

#### Description of the study populations

Six of the studies focused on Internal Medicine residents [26–30, 33]. One study included only Pediatric residents [25], and another Anesthesiology residents [31]. The studies from Mexico [34] and the Netherlands [32] did not focus on a specific specialty.

#### Measuring burnout

All of the 10 studies measured burnout using either the full Maslach Burnout Inventory (MBI) [2, 25–29, 32–34] or selected MBI sub-scales [30, 31]. The full MBI measures three dimensions of burnout: Emotional Exhaustion, Depersonalization and Personal Accomplishment. It is one of the most widely used measures of burnout in the scientific literature [35, 36].

#### Measuring quality of care

Three types of quality of care measures related to acceptability and safety were used in these studies. In terms of patient safety, they included medical errors/suboptimal care. For acceptability, they included perceived quality of care, and physician communication/attitudes.

#### Patient safety: Medical errors/suboptimal care

Eight studies assessed medical errors [25, 27, 29–34]. Four of the studies [31–34] either used or adapted a measure first employed by Shanafelt and colleagues [33]. Shanafelt et al.’s [33] measure is comprised of eight items and asks respondents to report on the frequency with which they provide suboptimal patient care and have suboptimal patient care attitudes. These items were used to create two summary measures: (1) suboptimal patient care practices at least monthly, and (2) suboptimal patient care practices at least weekly. In their paper, Shanafelt et al. [33] note that the psychometric properties of the measure have not been established. Block et al. [30] used items from a questionnaire but did not reference the source. However, the items that were listed in their paper appear to collect information similar to Shanafelt et al.’s [33] instrument.

In their two studies, West et al. [27, 29] used one question to self-report the occurrence of a medical error in the past three months. It is similar to a question employed by Shanafelt et al. [33] for their study of the relationship between physician burnout and medical errors.

In contrast to the other included studies, Fahrenkopf et al. [25] used chart and medical order abstraction to collect practice data. Trained reviewers identified and categorized errors according to severity and preventability.

#### Acceptability: Perceived quality of care

One study [26] used six items from the Mini-Clinical Evaluation Exercise (CEX). The Mini-CEX was developed by the American Board of Internal Medicine [37] to assess resident clinical skills. In Beckman et al.’s [26] study, residents were evaluated by peers, senior residents, and non-physician professionals using the Mini-CEX. Two domains were studied: (1) desirability as a physician and (2) effective communication.

#### Acceptability: Communication/attitudes

Along with Beckman et al. [26], Passalacqua and Segrin [28] focused on physician communication. They adapted a 13-item patient-centered communication scale [38] that was designed to be used by nurses and patients to evaluate physician-centered communication. The adaptation involved making the scale a self-report measure.

### Relationship between burnout and safety and acceptability dimensions of quality of care

#### Safety dimension and burnout: Medical errors

There was a consistently significant relationship between burnout and medical errors found among the eight studies [25, 27, 29–34] that focused on this relationship (Table 2). de Oliveira et al. [31] observed a significant association between errors and risk of high burnout.

**Table 2:** Quality of Care Outcomes

Author(s)	Medical Errors (ME)	Quality of Care (QoC)	Communication/Attitudes
Beckman et al. (2012) [26] USA		Multivariate analysis outcome: Desirability as a physician (95% CI): Burnout Yes/No: Beta: 0.063 (−0.20, 0.08), *p* = 0.38 MBI-DP: Beta: −0.0006 (−0.01, 0.01), *p* = 0.93 MBI-EE: Beta: −0.0028 (−0.01, 0.004), *p* = 0.43 MBI-PA: Beta: −0.0032 (−0.01, 0.01), *p* = 0.47	Multivariate analysis outcome: Effective Communication (95% CI): Burnout Yes/No: Beta: 0.3046 (0.10, 0.51), *p* < 0.01 MBI-DP: Beta: 0.0239 (0.0066, 0.0412), *p* < 0.01 MBI-EE: Beta: 0.0090 (0.0004, 0.0177), *p* = 0.05 MBI-PA: Beta: 0.0020 (−0.01, 0.02), *p* = 0.81
Block et al. (2013) [30] USA	Make errors due to fatigue: High burnout: 88% Medium burnout: 88% Low burnout: 43% Make errors due to workload:* High burnout: 94% Medium burnout: 76% Low burnout: 67% Forget to order med: High burnout: 41% Medium burnout: 18% Low burnout: 14% Forget to convey info:* High burnout: 41% Medium burnout: 35% Low burnout: 14% *Significant difference across tertiles (p < 0.05)		
de Oliveira et al. (2013) [31] USA	Significant differences between high burnout risk only vs low burnout for all items: • I make mistakes without negative consequences to my patients • I perform procedures without appropriate training • I make mistakes with negative consequences to my patients • I fall short in the quality of care I provide to my patients • I do not have enough time or attention for my patients • I have made medication errors involving the wrong drug or dose in the last year • I do not monitor the patient in the operating room as closely as I should		
Fahrenkopf et al. (2008) [25] USA	Based on chart: Rates of error/resident month: Burnout = 0.45 (0.20, 0.98) No burnout = 0.53 (0.21, 1.33) *p* = 0.4 Self-report: Made significant medical error due to sleep deprivation: Burnout = 29% No burnout = 10% *p* = 0.05 Self-report: Mean number of errors over past month: Burnout = 2.3 No burnout = 1.0 *p* = 0.02		
Passalacqua and Segrin (2012) [28] USA			Correlation between patient-centered communication and burnout: r = −0.52, p < 0.001 Correlation between pre-post empathy decline and burnout: r = 0.28, *p* < 0.01
Prins et al. (2009) [32] The Netherlands	Correlation between: Moderate burnout and action/inexperience errors: r = 0.18, *p* < 0.001 Severe burnout and inaction/inexperience errors: r = 0.10, *p* < 0.001 Moderate burnout and errors due to lack of time: r = 0.36, *p* < 0.001 Severe burnout and errors due to lack of time: r = 0.23, *p* < 0.001		
Shanafelt et al. (2002) [33] USA	Odds Ratio of self-reported suboptimal patient care practiced monthly or weekly (95% CI): Lowest DP reference group 2nd Quintile DP 0.78 (0.19, 3.13), *p* > 0.20 3rd Quintile DP1.51 (0.45, 5.12), *p* > 0.20 4th Quintile DP4.00 (1.05, 15.21), *p* = 0.04 Highest DP 4.96 (1.39, 17.71), *p* = 0.01		
Toral-Villanueva et al. (2009) [34] Mexico	Odds Ratios of self-reported suboptimal patient care practiced monthly (95% CI): High EE: OR = 5.6 (2.9, 10.8) High DP: OR = 4.4 (2.3, 8.5) Low PA: OR = 2.1 (1.1, 4.0) With burnout: OR = 5.5 (2.7, 11.2), *p* < 0.001 Odds Ratios of self-reported suboptimal patient care practiced weekly (95% CI): High EE: OR = 8.3 (2.8, 24.3) High DP: OR = 3.3 (1.2, 8.9) With burnout: OR = 5.2 (1.6, 16.3), *p* = 0.005		
West et al. (2006) [27] USA	Association of burnout with a self-perceived major medical error in the following 3 months (95% CI): Controlling for depression and empathy: DP: OR = 1.10 (1.04, 1.16), *p* = 0.001 EE: OR = 1.07 (1.03, 1.12), *p* < 0.001 PA: OR = 0.93 (0.88, 0.99), *p* = 0.02		
West et al. (2009) [29] USA	Association of burnout with a self-perceived major medical error in the following 3 months (95% CI): Controlling for fatigue: DP: OR = 1.08 (1.04, 1.11), *p* < 0.001 EE: OR = 1.05 (1.03, 1.07), *p* < 0.001 PA: OR = 0.95 (0.92, 0.97), *p* < 0.001 Association of burnout with a self-perceived major medical error in the following 3 months (95% CI): Controlling for sleepiness: DP: OR = 1.05 (0.98, 1.12), *p* = 0.15 EE: OR = 1.07 (1.03, 1.10), *p* < 0.001 PA: OR = 0.94 (0.89, 0.99), *p* = 0.02		

Shanafelt et al. [33] observed a significant positive association between suboptimal patient care practiced monthly or weekly and DP. In addition, they reported a dose-response relationship such that as DP increased, so did the probability of suboptimal patient care. Prins et al. [32] reported significant correlations between moderate and severe burnout with action/inexperience errors; similarly, there were significant correlations between moderate and severe burnout with errors due to lack of time. Examining the MBI’s three sub-domains, Toral-Villanueva et al. [34] found that high EE and high DP increased the probabilities of self-reported patient care practiced both monthly and weekly. Using longitudinal data, West et al. [27, 29] observed similar results when controlling for depression, empathy, and fatigue. However, the relationship between self-perceived medical error and DP was no longer significant when the analysis controlled for sleepiness.

In their study, Block et al. [30] found a significant difference between severity of burnout and two error-related behaviors: (1) making errors due to workload and (2) forgetting to convey information. Mirroring the dose-response Shanafelt et al. [33] reported, Block et al. [30] saw the proportion of residents reporting these two behaviors increase as the severity of burnout increased.

Fahrenkopf et al. [25] found that there was a significant association between presence of burnout (versus burnout not present) and residents reporting a significant medical error due to sleep deprivation. In addition, respondents with burnout as opposed to those without it, reported a greater number of errors over the past month. However, when they used a measure based on reviews of the clinical records to assess medical errors, they did not find a significant difference in the groups with and without burnout.

#### Acceptability dimension: Perceived quality of care

There was one study that examined the relationship between burnout and perceived quality of care [26]. In this study, quality of care was evaluated by peers, senior residents, and non-physician professionals. There was no significant relationship found between burnout (i.e., any of the burnout sub-scales or total burnout score) and perceived desirability as a clinician.

#### Acceptability dimension: Communication/attitudes

There were two studies that examined the association between burnout and communication/attitudes. Beckman et al. [26] found a significant relationship between effective communication and DP as well as their summary burnout score. Passalacqua and Segrin [28] reported a significant negative correlation between patient-centered communication and burnout. In addition, they observed a positive correlation between empathy decline and burnout.

## Discussion

This systematic literature review identified 10 studies; seven of them were of moderate risk of bias and three of high risk of bias. In terms of quality of care reflecting patient safety, outcomes were measured with a focus on medical errors. The quality of care outcomes reflecting acceptability were measured with perceived quality of care and communication/attitudes. Eight of the studies examined the relationship between burnout and medical errors.

The results of the included studies suggest there is moderate evidence that burnout is associated with patient safety as reflected in self-perceived medical errors and sub-optimal care. However, the evidence for an association between burnout and acceptability aspects of quality is limited partly because there were few studies that focused on it.

These results have implications for the training milieu. Residents supervise medical students and serve as role models for these students. If burnout affects performance, resident burnout and errors can undermine the confidence of the students who residents supervise. In this way, resident burnout can impact medical student learning. Residents are still in training, and at the same time are asked to teach students, thus the importance for medical educators to address resident burnout is essential.

Moreover, for residents experiencing burnout, increased medical errors could amplify feelings of burnout [27]. This has led West et al. [27] to advocate for more interventions to prevent medical errors. The relationship between medical errors and burnout also suggests a need for more evidence-based interventions that support residents when they make errors as well as those that teach effective coping strategies [27]. Hospital and residency programs also could benefit from developing more formal mechanisms for processing medical errors not only to improve patient outcomes, but also physician well-being [39].

### Strengths and limitations of interpreting the literature

All of the studies used either the MBI or a variation of the MBI. This raises the question of the validity and comparability of the full MBI versus selected items from it. For example, Block et al. [30] used six of the 22 items and de Oliveira et al. [31] incorporated 12 of the items into their study. It would be helpful to interpreting the effects of burnout if there were additional studies examining the psychometric properties of the abbreviated versions of the MBI.

In addition, the studies relied on resident self-report data to assess medical errors. The self-report could be influenced by a number of factors including recall bias, social desirability, and the influence of burnout. For example, Fahrenkopf et al. [25] observed a discrepancy between the results of chart audits and resident self-report. Residents with higher burnout scores reported higher numbers of medical errors than the chart audits. There was no difference between groups with and without burnout in terms of medical errors identified through chart review. It is difficult to determine whether this was because there are differences in resident versus chart review definitions of errors. Further exploration would be useful to understand how residents view errors and understand their severity levels.

It will also be important to understand the nature of the relationship between burnout and the reporting of errors. For example, does the incongruity between self-report and chart audits reflect a decreased confidence in skills? Residents are often placed in situations where they are asked to perform independently, but because they are also still learners, they may not feel adequately prepared. Concurrent experiences of burnout with perceived errors could exacerbate feelings of being an imposter. Or, is the association between burnout and errors a reflection of a relationship between burnout and decreased cognitive functioning [9]? If this is the case, this relationship may be highlighting burnout’s interference with clinical skill acquisition. Given that residents are still in training, it is important for medical educators to understanding the consequences of burnout and its relationship to these intermediate outcomes (i.e., decreased confidence and increased risk of cognitive errors). This knowledge can be used to modify training structures and approaches as well as to develop effective supports that also address these types of intermediate outcomes to promote optimal learning environments.

Another limitation of the existing body of literature is the reliance on cross-sectional study designs. Cross-sectional design prevents conclusions with regard to causality. This is because cross-sectional data does not reflect the sequence of events. That is, what was first – burnout or the outcome? Did burnout cause decreased quality of care? Or, did decreased quality of care cause burnout? Analyses based on cross-sectional data limit the extent to which conclusions about relationships between burnout and quality can be drawn. They cannot be used to determine the causal nature of the relationship between the two. However, three of the studies used longitudinal data and their findings suggest that burnout leads to medical errors [29] and decreased empathy [28] among residents. Additional longitudinal research about which factors contribute or protect residents from burnout and medical errors would be useful in developing training phase specific interventions. Also, research regarding the role of burnout would be useful. For example, is burnout a moderator of sleep deprivation leading to increased risk of medical errors? Or, does sleep deprivation moderate burnout and lead to an increased risk of medical errors?

Finally, two studies [26, 27] tested for a difference in the characteristics of study participants and non-participants. This information is helpful in interpreting the generalizability of the results. It would be helpful for interpreting results if future studies included this type of information in their reports.

### Strengths and limitations of the search strategy

One of the search limitations is related to the databases used. If articles did not appear in any of our search databases, they would have been missed. To minimize this limitation, five databases were searched. In addition, we employed inclusive search terms for each database and employed a hand search of included articles. Another potential limitation is the fact that the search focused on articles published in English-language journals. It is interesting that there are studies about physician burnout and quality of care that are reported in English-language journals although the studies were conducted in Europe, the Middle East, North America and Asia. In this review, we found few non-US articles that specifically focus on resident burnout and quality of care. An important follow-up question is whether research about burnout among residents and quality of care is uniquely relevant to the US. If this is the case, is it because resident training programs in other countries have fewer opportunities for patient contact? Or, is the reason there are few English-language publications because physician training is country specific and, as a result, these types of papers are published in the native languages of the researchers?

## Conclusions

The results of this systematic literature review suggest there is moderate evidence that burnout is associated with patient safety. Because resident burnout has important implications for quality of care and training, it is an important consideration in medical education. In addition, these results suggest that future research evaluating burnout interventions for residents could consider looking at changes on patient safety to assess the effectiveness of burnout interventions.

There are relatively fewer studies looking at the relationship between resident burnout and patient acceptability-related quality of care. The existing studies suggest there could be a relationship between burnout and patient acceptability-related quality of care. This body of literature could be improved if more attention was to be given to acceptability and the development of psychometrically sound measures of it. If the physician-patient collaboration truly is one of the critical interactions in healthcare, greater attention should be paid to its measurement and enhancement.

## Additional files

Additional file 1: PRISMA checklist. A 27-item checklist pertaining to the content of a systematic review (PDF 77 kb)

Additional file 2: Search strategy and keywords. Database specific search strategies and keywords used (PDF 33 kb)

Additional file 3: Risk of bias assessment checklist. Summary of risk of bias assessment results within accepted studies (PDF 16 kb)

## Abbreviations

CEX: Mini-Clinical Evaluation Exercise
DP: Depersonalization
EE: Emotional Exhaustion
IOM: Institute of Medication
MBI: Maslach Burnout Inventory
MERSQI: Medical Education Research Study Quality Instrument
PA: Personal Accomplishment
PRESS: Peer Review of Electronic Search Strategies
PRISMA: The Preferred Reporting Items for Systematic Reviews and Meta-Analyses
STROBE: Strengthening of Observational Studies in Epidemiology
WHO: World Health Organization
