# Supplementary material for: The relationship between resident burnout and safety-related and acceptability-related quality of healthcare: a systematic literature review
Source: BMC Med Educ. 2017 Nov 9;17:195. doi: 10.1186/s12909-017-1040-y (PMC5680598; doi:10.1186/s12909-017-1040-y)
Supplement: Supplementary file 2 — Search strategy and keywords. Database specific search strategies and keywords used (PDF 33 kb) [file 12909_2017_1040_MOESM2_ESM.pdf]

Search Strategy

Database**:** Medline Current

Search Terms**:** [exp Burnout, Professional/ OR burnout.mp. OR (burnout adj3 effect$).mp.] **AND** [residen$ OR intern$ OR PGY$ OR post$ graduat$ OR exp Physicians/ OR exp Psychiatry/ OR allergist$.mp. OR anesthesiologist$.mp. OR cardiologist$.mp. OR clinical pharmacologist$.mp. OR clinical toxicologist$.mp. OR dermatologist$.mp. OR doctor$.mp. OR endocrinologist$.mp. OR gastroenterologist$.mp. OR gynecologist$.mp. OR hematologist$.mp. OR immunologist$.mp. OR medical biochemist$.mp. OR medical geneticist$.mp. OR medical microbiologist$.mp. OR nephrologist$.mp. OR neurologist$.mp. OR neuropathologist$.mp. OR neuroradiologist$.mp. OR occupational physician$.mp. OR oncologist$.mp. OR ophthalmologist$.mp. OR pathologist$.mp. OR pediatrician$.mp. OR physician$.mp. OR psychiatrist$.mp. OR radiologist$.mp. OR rheumatologist$.mp. OR surgeon$.mp. OR urologist$.mp.] **AND** [exp Diagnostic Errors/ OR exp Medical Errors/ OR exp Medication Errors/ OR exp "Quality of Health Care"/ OR exp Quality Assurance, Health Care/ OR misdiag$.mp. OR (diagnos$ adj3 error$).mp. OR (medical$ adj3 error$).mp. OR (medication$ adj3 error$).mp. OR (drug$ adj3 error$).mp. OR (mistak$ adj3 medic$).mp. OR (surgic$ adj3 error$).mp. OR (quality$ adj3 health$ adj3 care$).mp. OR (quality$ adj3 healthcare$).mp. OR (quality$ adj3 of adj3 care$).mp. OR exp Professional Competence/ OR (professional$ adj3 competenc$).mp. OR (technical$ adj3 expertise$).mp. OR (expertise$ adj3 generaliz$).mp. OR professionalism$.mp. OR exp Treatment Outcome/ OR (treat$ adj3

outcome$).mp. OR (patient$ adj3 outcome$).mp. OR exp Professional Impairment/ OR (impair$ adj3 physician$).mp. OR (impair$ adj3 doctor$).mp. OR (disruptive$ adj3 behav$).mp. OR exp Safety/ OR safe$.mp. OR exp Risk/ OR risk$.mp. OR exp Patient Satisfaction/ OR (patient$ adj3 satisf$).mp. OR (client$ adj3 satisf$).mp. OR exp Professional-Patient Relations/ OR (professional$ adj3 patient$ adj3 relation$).mp. OR (client$ adj3 contact$).mp. OR exp Physician-Patient Relations/ OR (physician$ adj3 patient$ adj3 relation$).mp. OR (doctor$ adj3 patient$ adj3 relation$).mp. OR exp Communication/ OR communicat$.mp. OR misinform$.mp. OR exp Health Communication/ OR exp "Attitude of Health Personnel"/ OR attitude$.mp. OR exp Clinical Competence/ OR (clinical$ adj3 competenc$).mp. OR (clinical$ adj3 skill$).mp. OR exp Patient-Centered Care/ OR (patient$ adj3 cent$ adj3 care$).mp. OR (patient$ adj3 focus$ adj3 care$).mp. OR exp Empathy/ OR empath$.mp. OR exp Patient Care/ OR (patient$ adj3 care$).mp. OR (informal$ adj3 care$).mp. OR exp "Standard of Care"/ OR (standard$ adj3 care$).mp. OR st.fs. [standards - floating subheading] OR exp Self Efficacy/ OR efficacy$.mp. OR exp Clinical Audit/ OR audit$.mp. OR exp Medical Audit/ OR (diagnos$ adj3 mistak$).mp. OR (medication$ adj3 mistak$).mp. OR (drug$ adj3 mistak$).mp. OR (surgic$ adj3 mistak$).mp. OR exp Safety Management/ OR (program$ adj3 hazard$ adj3 surveillance$).mp. OR (management$ adj3 safety$).mp. OR (hazard$ adj3 control$).mp. OR (hazard$ adj3 management$).mp. OR exp Malpractice/ OR malpractic$.mp. OR negligen$.mp. OR exp Morbidity/ OR morbidit$.mp. OR exp Postoperative Complications/ OR (postoperative$ adj3 complication$).mp. OR exp Cross Infection/ OR (nosocomial$ adj3 infection$).mp. OR (hospital$ adj3 infection$).mp. OR (cross$ adj3 infection$).mp. OR exp Physician's

Practice Patterns/ OR (practice$ adj3 pattern$ adj3 clinical$).mp. OR (practice$ adj3 pattern$ adj3 physician$).mp. OR (prescribing$ adj3 pattern$ adj3 physician$).mp. OR (practice$ adj3 pattern$ adj3 professional$).mp. OR (practice$ adj3 pattern$ adj3 variation$).mp. OR (practice$ adj3 clinical$ adj3 variation$).mp. OR (practice$ adj3 medical$ adj3 variation$).mp. OR exp Mortality/ OR (rate$ adj3 age-specific$ adj3 death$).mp. OR (rate$ adj3 death$).mp. OR (rate$ adj3 fatalit$).mp. OR mortalit$.mp. OR exp "Outcome Assessment (Health Care)"/ OR (measure$ adj3 outcome$).mp. OR (assessment$ adj3 outcome$).mp. OR (research$ adj3 outcome$).mp. OR (stud$ adj3 outcome$).mp. OR (assessment$ adj3 patient$ adj3 outcome$).mp. OR (research$ adj3 patient$ adj3 outcome$).mp. OR exp Risk Reduction Behavior/ OR exp Risk-Taking/ OR exp "Root Cause Analysis"/ OR (cause$ adj3 root$ adj3 analys$).mp. OR exp "Drug-Related Side Effects and Adverse Reactions"/ OR (drug$ adj3 side$ adj3 effect$).mp. OR (drug$ adj3 toxic$).mp. OR (drug$ adj3 reaction$ adj3 adverse$).mp. OR (drug$ adj3 event$ adj3 adverse$).mp. OR ae.fs. [adverse effects floating subheading] OR mo.fs. [mortality floating subheading] OR po.fs. [poisoning floating subheading] OR to.fs. [toxicity floating subheading] OR in.fs. [injuries floating subheading]]

Database: Medline In-Process

Search Terms: [exp Burnout, Professional/ OR burnout.mp. OR (burnout adj3 effect$).mp.] **AND** [residen$ OR intern$ OR PGY$ OR post$ graduat$ OR exp Physicians/ OR exp Psychiatry/ OR allergist$.mp. OR anesthesiologist$.mp. OR cardiologist$.mp. OR clinical pharmacologist$.mp. OR clinical toxicologist$.mp. OR dermatologist$.mp. OR doctor$.mp. OR endocrinologist$.mp. OR

gastroenterologist$.mp. OR gynecologist$.mp. OR hematologist$.mp. OR immunologist$.mp. OR medical biochemist$.mp. OR medical geneticist$.mp. OR medical microbiologist$.mp. OR nephrologist$.mp. OR neurologist$.mp. OR neuropathologist$.mp. OR neuroradiologist$.mp. OR occupational physician$.mp. OR oncologist$.mp. OR ophthalmologist$.mp. OR pathologist$.mp. OR pediatrician$.mp. OR physician$.mp. OR psychiatrist$.mp. OR radiologist$.mp. OR rheumatologist$.mp. OR surgeon$.mp. OR urologist$.mp.] **AND** [exp Diagnostic Errors/ OR exp Medical Errors/ OR exp Medication Errors/ OR exp "Quality of Health Care"/ OR exp Quality Assurance, Health Care/ OR misdiag$.mp. OR (diagnos$ adj3 error$).mp. OR (medical$ adj3 error$).mp. OR (medication$ adj3 error$).mp. OR (drug$ adj3 error$).mp. OR (mistak$ adj3 medic$).mp. OR (surgic$ adj3 error$).mp. OR (quality$ adj3 health$ adj3 care$).mp. OR (quality$ adj3 healthcare$).mp. OR (quality$ adj3 of adj3 care$).mp. OR exp Professional Competence/ OR (professional$ adj3 competenc$).mp. OR (technical$ adj3 expertise$).mp. OR (expertise$ adj3 generaliz$).mp. OR professionalism$.mp. OR exp Treatment Outcome/ OR (treat$ adj3 outcome$).mp. OR (patient$ adj3 outcome$).mp. OR exp Professional Impairment/ OR (impair$ adj3 physician$).mp. OR (impair$ adj3 doctor$).mp. OR (disruptive$ adj3 behav$).mp. OR exp Safety/ OR safe$.mp. OR exp Risk/ OR risk$.mp. OR exp Patient Satisfaction/ OR (patient$ adj3 satisf$).mp. OR (client$ adj3 satisf$).mp. OR exp Professional-Patient Relations/ OR (professional$ adj3 patient$ adj3 relation$).mp. OR (client$ adj3 contact$).mp. OR exp Physician-Patient Relations/ OR (physician$ adj3 patient$ adj3 relation$).mp. OR (doctor$ adj3 patient$ adj3 relation$).mp. OR exp Communication/ OR communicat$.mp. OR misinform$.mp. OR exp Health

Communication/ OR exp "Attitude of Health Personnel"/ OR attitude$.mp. OR exp Clinical Competence/ OR (clinical$ adj3 competenc$).mp. OR (clinical$ adj3 skill$).mp. OR exp Patient-Centered Care/ OR (patient$ adj3 cent$ adj3 care$).mp. OR (patient$ adj3 focus$ adj3 care$).mp. OR exp Empathy/ OR empath$.mp. OR exp Patient Care/ OR (patient$ adj3 care$).mp. OR (informal$ adj3 care$).mp. OR exp "Standard of Care"/ OR (standard$ adj3 care$).mp. OR st.fs. [standards - floating subheading] OR exp Self Efficacy/ OR efficacy$.mp. OR exp Clinical Audit/ OR audit$.mp. OR exp Medical Audit/ OR (diagnos$ adj3 mistak$).mp. OR (medication$ adj3 mistak$).mp. OR (drug$ adj3 mistak$).mp. OR (surgic$ adj3 mistak$).mp. OR exp Safety Management/ OR (program$ adj3 hazard$ adj3 surveillance$).mp. OR (management$ adj3 safety$).mp. OR (hazard$ adj3 control$).mp. OR (hazard$ adj3 management$).mp. OR exp Malpractice/ OR malpractic$.mp. OR negligen$.mp. OR exp Morbidity/ OR morbidit$.mp. OR exp Postoperative Complications/ OR (postoperative$ adj3 complication$).mp. OR exp Cross Infection/ OR (nosocomial$ adj3 infection$).mp. OR (hospital$ adj3 infection$).mp. OR (cross$ adj3 infection$).mp. OR exp Physician's Practice Patterns/ OR (practice$ adj3 pattern$ adj3 clinical$).mp. OR (practice$ adj3 pattern$ adj3 physician$).mp. OR (prescribing$ adj3 pattern$ adj3 physician$).mp. OR (practice$ adj3 pattern$ adj3 professional$).mp. OR (practice$ adj3 pattern$ adj3 variation$).mp. OR (practice$ adj3 clinical$ adj3 variation$).mp. OR (practice$ adj3 medical$ adj3 variation$).mp. OR exp Mortality/ OR (rate$ adj3 age-specific$ adj3 death$).mp. OR (rate$ adj3 death$).mp. OR (rate$ adj3 fatalit$).mp. OR mortalit$.mp. OR exp "Outcome Assessment (Health Care)"/ OR (measure$ adj3 outcome$).mp. OR

(assessment$ adj3 outcome$).mp. OR (research$ adj3 outcome$).mp. OR (stud$ adj3 outcome$).mp. OR (assessment$ adj3 patient$ adj3 outcome$).mp. OR (research$ adj3 patient$ adj3 outcome$).mp. OR exp Risk Reduction Behavior/ OR exp Risk-Taking/ OR exp "Root Cause Analysis"/ OR (cause$ adj3 root$ adj3 analys$).mp. OR exp "Drug-Related Side Effects and Adverse Reactions"/ OR (drug$ adj3 side$ adj3 effect$).mp. OR (drug$ adj3 toxic$).mp. OR (drug$ adj3 reaction$ adj3 adverse$).mp. OR (drug$ adj3 event$ adj3 adverse$).mp. OR ae.fs. [adverse effects floating subheading] OR mo.fs. [mortality floating subheading] OR po.fs. [poisoning floating subheading] OR to.fs. [toxicity floating subheading] OR in.fs. [injuries floating subheading]]

Database**:** PsycINFO

Search Terms**:** [burnout.mp. OR (burnout adj3 effect$).mp.] **AND** [residen$ OR intern$ OR PGY$ OR post$ graduat$ OR exp physicians/ OR exp clinicians/ OR exp Psychiatry/ OR allergist$.mp. OR anesthesiologist$.mp. OR cardiologist$.mp. OR clinical pharmacologist$.mp. OR clinical toxicologist$.mp. OR dermatologist$.mp. OR doctor$.mp. OR endocrinologist$.mp. OR gastroenterologist$.mp. OR gynecologist$.mp. OR hematologist$.mp. OR immunologist$.mp. OR medical biochemist$.mp. OR medical geneticist$.mp. OR medical microbiologist$.mp. OR nephrologist$.mp. OR neurologist$.mp. OR neuropathologist$.mp. OR neuroradiologist$.mp. OR occupational physician$.mp. OR oncologist$.mp. OR ophthalmologist$.mp. OR pathologist$.mp. OR pediatrician$.mp. OR physician$.mp. OR psychiatrist$.mp. OR radiologist$.mp. OR rheumatologist$.mp. OR surgeon$.mp.

OR urologist$.mp.] **AND** [exp Errors/ OR exp "Quality of Care"/ OR misdiag$.mp. OR (diagnos$ adj3 error$).mp. OR (medical$ adj3 error$).mp. OR (medication$ adj3 error$).mp. OR (drug$ adj3 error$).mp. OR (mistak$ adj3 medic$).mp. OR (surgic$ adj3 error$).mp. OR (quality$ adj3 health$ adj3 care$).mp. OR (quality$ adj3 healthcare$).mp. OR (quality$ adj3 of adj3 care$).mp. OR exp Professional Competence/ OR (professional$ adj3 competenc$).mp. OR (technical$ adj3 expertise$).mp. OR (expertise$ adj3 generaliz$).mp. OR professionalism$.mp. OR exp Treatment Outcome/ OR (treat$ adj3 outcome$).mp. OR (patient$ adj3 outcome$).mp. OR exp Impaired Professionals/ OR (impair$ adj3 physician$).mp. OR (impair$ adj3 doctor$).mp. OR (disruptive$ adj3 behav$).mp. OR exp Safety/ OR safe$.mp. OR exp Risk Factors/ OR exp Risk Management/ OR exp Risk Assessment/ OR risk$.mp. OR exp Client Satisfaction/ OR (patient$ adj3 satisf$).mp. OR (client$ adj3 satisf$).mp. OR exp Therapeutic Processes/ OR (professional$ adj3 patient$ adj3 relation$).mp. OR (client$ adj3 contact$).mp. OR (physician$ adj3 patient$ adj3 relation$).mp. OR (doctor$ adj3 patient$ adj3 relation$).mp. OR exp Communication/ OR communicat$.mp. OR misinform$.mp. OR exp Communication Skills/

OR exp Communication Barriers/ OR exp Health Personnel Attitudes/ OR attitude$.mp. OR exp Competence/ OR (clinical$ adj3 competenc$).mp. OR (clinical$ adj3 skill$).mp. OR exp Client Centered Therapy/ OR (patient$ adj3 cent$ adj3 care$).mp. OR (patient$ adj3 focus$ adj3 care$).mp. OR exp Empathy/ OR empath$.mp. OR exp Patients/ OR (patient$ adj3 care$).mp. OR (informal$ adj3 care$).mp. OR exp Professional Standards/ OR (standard$ adj3 care$).mp. OR exp Self Efficacy/ OR efficacy$.mp. OR exp Clinical Audits/ OR audit$.mp. OR

(diagnos$ adj3 mistak$).mp. OR (medication$ adj3 mistak$).mp. OR (drug$ adj3 mistak$).mp. OR (surgic$ adj3 mistak$).mp. OR (program$ adj3 hazard$ adj3 surveillance$).mp. OR (management$ adj3 safety$).mp. OR (hazard$ adj3 control$).mp. OR (hazard$ adj3 management$).mp. OR exp Professional Liability/ OR malpractic$.mp. OR negligen$.mp. OR exp Morbidity/ OR morbidit$.mp. OR exp Postoperative Complications/ OR (postoperative$ adj3 complication$).mp. OR (nosocomial$ adj3 infection$).mp. OR (hospital$ adj3 infection$).mp. OR (cross$ adj3 infection$).mp. OR exp Clinical Practice/ OR (practice$ adj3 pattern$ adj3 clinical$).mp. OR (practice$ adj3 pattern$ adj3 physician$).mp. OR (prescribing$ adj3 pattern$ adj3 physician$).mp. OR (practice$ adj3 pattern$ adj3 professional$).mp. OR (practice$ adj3 pattern$ adj3 variation$).mp. OR (practice$ adj3 clinical$ adj3 variation$).mp. OR (practice$ adj3 medical$ adj3 variation$).mp. OR exp Mortality Rate/ OR exp "Death and Dying"/ OR (rate$ adj3 age-specific$ adj3 death$).mp. OR (rate$ adj3 death$).mp. OR (rate$ adj3 fatalit$).mp. OR mortalit$.mp. OR exp Treatment Effectiveness Evaluation/ OR (measure$ adj3 outcome$).mp. OR (assessment$ adj3 outcome$).mp. OR (research$ adj3 outcome$).mp. OR (stud$ adj3 outcome$).mp. OR (assessment$ adj3 patient$ adj3 outcome$).mp. OR (research$ adj3 patient$ adj3 outcome$).mp. OR exp Risk-Taking/ OR exp Error Analysis/ OR (cause$ adj3 root$ adj3 analys$).mp. OR exp "Side Effects (Drug)"/ OR (drug$ adj3 side$ adj3 effect$).mp. OR (drug$ adj3 toxic$).mp. OR (drug$ adj3 reaction$ adj3 adverse$).mp. OR (drug$ adj3 event$ adj3 adverse$).mp. OR exp Toxic Disorders/ OR exp Injuries/ OR 3620.cc. [Personnel Management & Selection & Training classification code] OR 3630.cc. [Personnel Evaluation & Job Performance classification code] OR 3650.cc. [Personnel Attitudes &

Job Satisfaction classification code] OR 3670.cc. [Working Conditions & Industrial Safety classification code] OR 3430.cc. [Professional Personnel Attitudes & Characteristics classification code] OR 3450.cc. [Professional Ethics & Standards & Liability code] OR 3470.cc. [Impaired Professionals classification code]]

Database**:** Embase

Search Terms**:** [exp Burnout/ OR burnout.mp. OR (burnout adj3 effect$).mp.] **AND** [residen$ OR intern$ OR PGY$ OR post$ graduat$ OR exp Physicians/ OR exp Psychiatry/ OR allergist$.mp. OR anesthesiologist$.mp. OR cardiologist$.mp. OR clinical pharmacologist$.mp. OR clinical toxicologist$.mp. OR dermatologist$.mp. OR doctor$.mp. OR endocrinologist$.mp. OR gastroenterologist$.mp. OR gynecologist$.mp. OR hematologist$.mp. OR immunologist$.mp. OR medical biochemist$.mp. OR medical geneticist$.mp. OR medical microbiologist$.mp. OR nephrologist$.mp. OR neurologist$.mp. OR neuropathologist$.mp. OR neuroradiologist$.mp. OR occupational physician$.mp. OR oncologist$.mp. OR ophthalmologist$.mp. OR pathologist$.mp. OR pediatrician$.mp. OR physician$.mp. OR psychiatrist$.mp. OR radiologist$.mp. OR rheumatologist$.mp. OR surgeon$.mp. OR urologist$.mp.] **AND** [exp Diagnostic Errors/ OR exp Medical Errors/ OR exp Medication Errors/ OR exp Health care quality/ OR exp Quality control/ OR misdiag$.mp. OR (diagnos$ adj3 error$).mp. OR (medical$ adj3 error$).mp. OR (medication$ adj3 error$).mp. OR (drug$ adj3 error$).mp. OR (mistak$ adj3 medic$).mp. OR (surgic$ adj3 error$).mp. OR (quality$ adj3 health$ adj3 care$).mp. OR (quality$ adj3 healthcare$).mp. OR (quality$ adj3 of adj3 care$).mp. OR exp

Professional Competence/ OR (professional$ adj3 competenc$).mp. OR (technical$ adj3 expertise$).mp. OR (expertise$ adj3 generaliz$).mp. OR professionalism$.mp. OR exp Treatment Outcome/ OR (treat$ adj3 outcome$).mp. OR (patient$ adj3 outcome$).mp. OR exp Malpractice/ OR (impair$ adj3 physician$).mp. OR (impair$ adj3 doctor$).mp. OR (disruptive$ adj3 behav$).mp. OR exp Safety/ OR safe$.mp. OR exp Risk OR exp Risk Factors/ OR exp Risk Assessment/ OR exp Risk Management/ OR risk$.mp. OR exp patient satisfaction/ OR (patient$ adj3 satisf$).mp. OR (client$ adj3 satisf$).mp. OR exp human relation/ OR (professional$ adj3 patient$ adj3 relation$).mp. OR (client$ adj3 contact$).mp. OR exp doctor patient relation/ OR (physician$ adj3 patient$ adj3 relation$).mp. OR (doctor$ adj3 patient$ adj3 relation$).mp. OR exp interpersonal communication/ OR communicat$.mp. OR misinform$.mp. OR exp communication disorder/ OR exp communication skill/ OR exp health personnel attitude/ OR attitude$.mp. OR exp clinical competence/ OR (clinical$ adj3 competenc$).mp. OR (clinical$ adj3 skill$).mp. OR exp patient care/ OR (patient$ adj3 cent$ adj3 care$).mp. OR (patient$ adj3 focus$ adj3 care$).mp. OR exp Empathy/ OR empath$.mp. OR exp medical care/ OR (patient$ adj3 care$).mp. OR (informal$ adj3 care$).mp. OR exp professional standard/ OR (standard$ adj3 care$).mp. OR exp standard/ OR exp Self Efficacy/ OR efficacy$.mp. OR exp medical audit/ OR audit$.mp. OR (diagnos$ adj3 mistak$).mp. OR (medication$ adj3 mistak$).mp. OR (drug$ adj3 mistak$).mp. OR (surgic$ adj3 mistak$).mp. OR (program$ adj3 hazard$ adj3 surveillance$).mp. OR (management$ adj3 safety$).mp. OR (hazard$ adj3 control$).mp. OR (hazard$ adj3 management$).mp. OR malpractic$.mp. OR negligen$.mp. OR exp Morbidity/ OR morbidit$.mp. OR exp postoperative complication/

OR (postoperative$ adj3 complication$).mp. OR exp Cross Infection/ OR (nosocomial$ adj3 infection$).mp. OR (hospital$ adj3 infection$).mp. OR (cross$ adj3 infection$).mp. OR exp clinical practice/ OR exp professional practice/ OR (practice$ adj3 pattern$ adj3 clinical$).mp. OR (practice$ adj3 pattern$ adj3 physician$).mp. OR (prescribing$ adj3 pattern$ adj3 physician$).mp. OR (practice$ adj3 pattern$ adj3 professional$).mp. OR (practice$ adj3 pattern$ adj3 variation$).mp. OR (practice$ adj3 clinical$ adj3 variation$).mp. OR (practice$ adj3 medical$ adj3 variation$).mp. OR exp mortality/ OR exp death/ OR (rate$ adj3 age-specific$ adj3 death$).mp. OR (rate$ adj3 death$).mp. OR (rate$ adj3 fatalit$).mp. OR mortalit$.mp. OR exp outcome assessment/ OR (measure$ adj3 outcome$).mp. OR (assessment$ adj3 outcome$).mp. OR (research$ adj3 outcome$).mp. OR (stud$ adj3 outcome$).mp. OR (assessment$ adj3 patient$ adj3 outcome$).mp. OR (research$ adj3 patient$ adj3 outcome$).mp. OR exp risk reduction/ OR exp high risk behavior/ OR exp "root cause analysis"/ OR (cause$ adj3 root$ adj3 analys$).mp. OR exp adverse drug reaction/ OR (drug$ adj3 side$ adj3 effect$).mp. OR (drug$ adj3 toxic$).mp. OR (drug$ adj3 reaction$ adj3 adverse$).mp. OR (drug$ adj3 event$ adj3 adverse$).mp. OR ae.fs. [adverse drug reaction] OR to.fs. [drug toxicity] OR dt.fs. [drug interaction subheading] OR si.fs. [side effect subheading] OR co.fs. [complication subheading]]

<u>Database</u>**:** Web of Science

<u>Search Terms</u>**:** [burn out* OR burnout*] **AND** [residen* OR intern* OR PGY* OR post* graduat* OR physician* OR clinician* OR psychiatry* OR allergist* OR anesthesiologist* OR cardiologist* OR clinical pharmacologist* OR clinical toxicologist* OR dermatologist* OR doctor* OR endocrinologist* OR gastroenterologist* OR gynecologist* OR

hematologist* OR immunologist* OR medical biochemist* OR medical geneticist* OR medical microbiologist* OR nephrologist* OR neurologist* OR neuropathologist* OR neuroradiologist* OR occupational physician* OR oncologist* OR ophthalmologist* OR pathologist* OR pediatrician* OR physician* OR psychiatrist* OR radiologist* OR rheumatologist* OR surgeon* OR urologist* OR consultant*] **AND** [error* OR health* care*OR healthcare* OR quality* OR misdiag* OR mistak* OR competenc* OR expertis* OR professionalism* OR outcome* OR impair* OR disruptive* OR safe* OR risk* OR satisf* OR relation* OR contact* OR communicat* OR misinform* OR attitude* OR skill* OR care* OR empath* OR standard* OR audit* OR hazard* OR malpractic* OR negligen* OR morbidit* OR infection* OR practice* pattern* OR prescrib* pattern* OR mortalit* OR death* OR fatalit* OR drug* OR adverse* OR poison* OR toxic* OR injur*]
