# Supplementary material for: The relationship between resident burnout and safety-related and acceptability-related quality of healthcare: a systematic literature review
Source: BMC Med Educ. 2017 Nov 9;17:195. doi: 10.1186/s12909-017-1040-y (PMC5680598; doi:10.1186/s12909-017-1040-y)
Supplement: Supplementary file 3 — Risk of bias assessment checklist. Summary of risk of bias assessment results within accepted studies (PDF 16 kb) [file 12909_2017_1040_MOESM3_ESM.pdf]

Risk of Bias Assessment Checklist

Author(s)	1	2	3	4	5	6	7	8	9	Total Score
Beckman et al. (2011) [24]	1	0	1	1	1	1	1	1	0	7
Block et al. (2013) [28]	0	0	1	0	1	1	0	1	0	4
de Oliveira et al. (2013) [29]	0	1	1	0	1	1	0	1	0	5
Fahrenkopf et al. (2008) [23]	1	1	1	1	1	1	0	1	0	7
Passalacqua and Segrin (2012) [26]	0	0	0	0	1	1	0	1	1	4
Prins et al. (2009) [30]	0	1	0	0	1	1	0	1	0	4
Shanafelt et al. (2002) [31]	0	1	1	0	1	1	1	1	0	6
Toral-Villanueva et al. (2008) [32]	0	1	1	0	1	1	1	1	0	6
West et al. (2006) [25]	1	1	1	0	1	1	0	1	1	7
West et al. (2009) [27]	0	1	1	0	1	1	0	1	1	6

Risk of Bias Assessment Criteria

1. Study population is well described (e.g., age, sex, location of the study, physician specialty, practice location) or test for significant differences between respondents and non-respondents
2. Data collection methods that address the risk of bias are described
3. Participation/response rate was at least 50% on average
4. Used a validated outcome measure/process
5. Statistical method was appropriate for the question being answered
6. Statistical significance of associations were tested and reported
7. Study controlled for relevant confounding factors - at least one confounder such as sex or age was considered in the analyses
8. Resident matched with patient rather than matching the data from the Unit in which the resident was practicing and patients that were treated by the Unit
9. Longitudinal data was used
